# Association between sarcopenia and fall history in middle-aged and older adults: a multidimensional clinical analysis of nutritional status, bone health, and medication burden

**DOI:** 10.3389/fendo.2026.1906385

**Published:** 2026-08-12

**Authors:** Pin Wang, Jie Ke, Jingjing Wang, Jingyan Chen, Lei Zhang, Yan Yang

**Affiliations:** 1 Department of Endocrinology, Sichuan Provincial People’s Hospital, School of Medicine, University of Electronic Science and Technology of China, Chengdu, China; 2 Department of Endocrinology, School of Medicine, University of Electronic Science and Technology of China, Sichuan Provincial People’s Hospital, Chengdu, China

**Keywords:** calf circumference, fall history, falls, muscle mass, nutritional status, osteoporosis, polypharmacy, sarcopenia

## Abstract

**Background:**

Sarcopenia has been associated with an increased risk of falls in older adults; however, this association may be influenced by nutritional status, bone health, comorbidity burden, and medication use. This study aimed to investigate the association between sarcopenia and fall history in middle-aged and older adults and to further identify sarcopenia-related components associated with fall history.

**Methods:**

This cross-sectional clinical study included 298 middle-aged and older inpatients who underwent sarcopenia assessment, fall history collection, anthropometric measurements, nutritional assessment, laboratory testing, and bone health evaluation. Sarcopenia was diagnosed according to the Asian Working Group for Sarcopenia 2019 criteria. Fall history was defined as at least one self-reported fall in the past 12 months prior to assessment. Binary logistic regression models were used to evaluate the association between sarcopenia and fall history, with progressive adjustment for age, sex, BMI, number of comorbidities, polypharmacy, Mini Nutritional Assessment total score, and osteoporosis status. Associations between individual sarcopenia-related components and fall history were further examined.

**Results:**

Among the 298 participants, 125 were classified as having sarcopenia and 73 had a history of falls. Participants with sarcopenia had a higher prevalence of fall history than those without sarcopenia (32.0% vs. 19.1%, P = 0.010). In the fully adjusted model, sarcopenia remained significantly associated with fall history after adjustment for age, sex, BMI, comorbidity burden, polypharmacy, nutritional status, and osteoporosis (OR = 3.548, 95% CI 1.682–7.482, P = 0.001). In component analyses, lower appendicular skeletal muscle mass index (OR = 1.948 per 1 kg/m² decrease, 95% CI 1.190–3.191, P = 0.008), low muscle mass (OR = 2.799, 95% CI 1.467–5.340, P = 0.002), and smaller calf circumference (OR = 1.180 per 1 cm decrease, 95% CI 1.049–1.326, P = 0.006) were significantly associated with fall history. Handgrip strength, low handgrip strength, gait speed, and low gait speed were not significantly associated with fall history.

**Conclusion:**

Sarcopenia was significantly associated with fall history in middle-aged and older adults, independent of nutritional status, osteoporosis, comorbidity burden, and polypharmacy. Low muscle mass and smaller calf circumference were the sarcopenia-related components most clearly associated with fall history. These findings support the importance of evaluating sarcopenia-related fall susceptibility.

## 1 Introduction

Falls are common adverse health events in middle-aged and older adults and are associated with fractures, disability, hospitalization, loss of independence, reduced quality of life, and increased mortality. Rather than resulting from a single cause, falls usually arise from the interaction of multiple intrinsic and extrinsic factors, including impaired muscle strength and physical performance, gait and balance disorders, comorbidity burden, medication use, nutritional impairment, cognitive decline, and poor bone health. Current global guidelines therefore emphasize that fall-risk assessment and prevention should be based on a multidomain framework rather than on isolated risk factors alone (1).

Sarcopenia is a progressive skeletal muscle disorder characterized by low muscle mass, reduced muscle strength, and/or impaired physical performance. According to the Asian Working Group for Sarcopenia 2019 consensus, sarcopenia is diagnosed when low muscle mass is accompanied by low muscle strength or reduced physical performance (2). Because skeletal muscle plays a central role in mobility, postural control, and functional reserve, sarcopenia has been increasingly recognized as an important geriatric syndrome related to adverse outcomes, including falls, fractures, frailty, functional decline, and mortality. Previous systematic reviews and meta-analyses have shown that older adults with sarcopenia have a higher risk of falls and fractures than those without sarcopenia (3, 4).

However, the relationship between sarcopenia and falls may not be fully explained by muscle-related parameters alone. In clinical practice, individuals with sarcopenia often present with coexisting nutritional impairment, reduced body reserve, low albumin level, inflammation, decreased bone mineral density, multiple comorbidities, and medication burden. Poor nutritional status, as assessed by the Mini Nutritional Assessment, has been reported to be closely associated with sarcopenia in older adults (5). Similarly, polypharmacy and comorbidity burden are important components of fall-risk assessment, although medication burden may partly reflect underlying multimorbidity and overall frailty rather than acting as an isolated causal factor (6). These findings suggest that sarcopenia-related fall susceptibility should be interpreted within a broader clinical context that includes nutritional status, bone health, comorbidity burden, and medication use.

Although previous population-based studies have established an association between sarcopenia and falls, relatively fewer clinical studies have simultaneously evaluated sarcopenia-related measurements, nutritional status, bone mineral density, bone metabolism, comorbidity burden, and polypharmacy in relation to fall history. In addition, it remains unclear which components of sarcopenia, such as low muscle mass, handgrip strength, gait speed, or calf circumference, are most closely associated with fall history in clinical assessment settings. Therefore, the present study aimed to investigate the association between sarcopenia and fall history in middle-aged and older adults, and to further examine this association within a multidimensional clinical framework incorporating nutritional status, bone health, comorbidity burden, and medication burden.

## 2 Methods

### 2.1 Study design and participants

This study was a secondary analysis of pre-existing cross-sectional clinical survey data. The original study consecutively enrolled middle-aged and older inpatients from the Department of Endocrinology, Sichuan Academy of Medical Sciences and Sichuan Provincial People’s Hospital. All participants underwent sarcopenia-related assessment, fall history collection, anthropometric measurements, and relevant laboratory examinations. Based on the original dataset, the present study further used fall history as the primary outcome to investigate the association between sarcopenia and fall history, with additional consideration of nutritional status, bone health, comorbidity burden, and medication burden.

Participants were classified into the sarcopenia and non-sarcopenia groups according to the diagnostic criteria for sarcopenia. The inclusion criteria were as follows: middle-aged and older inpatients who met the age requirement of the original database; availability of sarcopenia classification and fall history records; ability to communicate and cooperate with handgrip strength, gait speed, and anthropometric measurements; and availability of muscle mass, nutritional status, or bone health assessments. Middle-aged and older adults were defined as those aged ≥45 years, consistent with the age threshold commonly used to define middle age in sarcopenia-related epidemiological studies in Asian populations; the youngest participant in the final analytic sample was 45 years old (mean age 69.3 ± 8.2 years, range 45–89 years). Of the 298 participants, 83 (27.9%) were aged 45–64 years (middle-aged) and 215 (72.1%) were aged ≥65 years (older adults).

Participants were excluded if they had limb amputation, a body mass index (BMI) >50 kg/m², a definite diagnosis of dementia, Parkinson’s disease, or other conditions that prevented completion of functional assessments, implanted electronic devices such as pacemakers, use of glucocorticoids, thyroid hormones, sex hormones, or other medications that could affect muscle, bone metabolism, or endocrine status within 3 months before enrollment, use of antibiotics or probiotics within 1 month before enrollment, infection, acute stress, dehydration, edema, or other clinical conditions during hospitalization that might affect measurements, participation in another clinical study, unwillingness to participate, or other conditions considered unsuitable by the investigators.

The study was approved by the Ethics Committee of Sichuan Academy of Medical Sciences and Sichuan Provincial People’s Hospital. Written informed consent was obtained from all participants.

### 2.2 Diagnosis of sarcopenia

Sarcopenia was diagnosed according to the Asian Working Group for Sarcopenia 2019 consensus (2). Sarcopenia was defined as low muscle mass accompanied by low muscle strength and/or reduced physical performance.

Appendicular skeletal muscle mass was measured using dual-energy X-ray absorptiometry (DXA), and appendicular skeletal muscle mass index (ASMI) was calculated as appendicular skeletal muscle mass divided by height squared:

ASMI = appendicular skeletal muscle mass (kg)/height² (m²).

Low muscle mass was defined as ASMI <7.0 kg/m² in men and <5.4 kg/m² in women. Muscle strength was assessed using handgrip strength. Low handgrip strength was defined as <28 kg in men and <18 kg in women. Physical performance was assessed using usual gait speed over a 6-meter walking test. Low gait speed was defined as gait speed <1.0 m/s.

#### 2.2.1 Definition of fall history

The primary outcome was fall history, defined as at least one self-reported fall in the past 12 months prior to assessment, consistent with the case-finding approach recommended by current fall-prevention guidelines (1). According to the original records, fall history was treated as a binary variable. Participants with no fall in the past 12 months were classified as having no fall history, whereas those who had experienced at least one fall in the past 12 months were classified as having fall history. If the original records further distinguished between single and recurrent falls within this 12-month window, these categories were combined into a single “fall history” category for the main analysis to ensure outcome stability.

### 2.3 Clinical data and medication assessment

Demographic and clinical data were collected, including age, sex, education level, smoking history, alcohol consumption, coffee or strong tea intake, parental history of fragility fracture, personal history of fragility fracture, height loss, pain score, number of comorbidities, medication use, calcium supplementation, vitamin D supplementation, and anti-osteoporosis medication use. Pain was assessed using a visual analogue scale. Polypharmacy was defined as the concurrent use of six or more medications.

#### 2.3.1 Anthropometric measurements

Height, weight, blood pressure, mid-upper arm circumference, and calf circumference were measured in all participants. Height and weight were measured twice after participants emptied their bladder, removed shoes and hats, and wore light clothing; the mean value was used for analysis. BMI was calculated as weight divided by height squared and expressed as kg/m².

Blood pressure was measured twice at the brachial artery in the seated resting position using an electronic sphygmomanometer, and the average value was recorded. Mid-upper arm circumference was measured at the thickest part of the upper arm while the arm was naturally relaxed. Calf circumference was measured around the thickest part of the right calf while participants stood with their feet shoulder-width apart.

#### 2.3.2 Assessment of muscle mass, muscle strength, and physical performance

Appendicular skeletal muscle mass was measured using a GE Healthcare-Lunar Prodigy Advance DXA scanner. ASMI was calculated as described above.

Handgrip strength was measured using an electronic hand dynamometer. Participants stood during the test and were instructed to squeeze the dynamometer with their dominant hand using maximal effort. The test was repeated three times, and the maximum value was used for analysis.

Physical performance was assessed using the 6-meter usual gait speed test. Participants were instructed to walk in a straight line at their usual pace, without walking aids whenever possible. The test was performed twice, and the average gait speed was calculated in meters per second.

#### 2.3.3 Bone mineral density and bone health assessment

Bone mineral density was measured using DXA at the femoral neck, total hip, and lumbar spine L1–L4. Osteoporosis was defined as a T-score ≤−2.5 at the femoral neck, total hip, or lumbar spine (8). Osteoporosis status and site-specific T-scores were included as bone health-related variables in the analysis.

#### 2.3.4 Laboratory measurements

After an overnight fast of at least 8 hours, venous blood samples were collected at approximately 8:00 a.m. and analyzed at the clinical laboratory of Sichuan Provincial People’s Hospital. Laboratory measurements included blood routine parameters, C-reactive protein, liver and kidney function, lipid profile, fasting plasma glucose, fasting insulin, fasting C-peptide, glycated hemoglobin, bone turnover markers, sex hormones, thyroid function, adrenocorticotropic hormone, morning cortisol, parathyroid hormone, and insulin-like growth factor-1.

Variables relevant to the present analysis included hemoglobin, C-reactive protein, total protein, albumin, 25-hydroxyvitamin D, serum calcium, serum phosphate, alkaline phosphatase, parathyroid hormone, β-C-terminal telopeptide of type I collagen, procollagen type I N-terminal propeptide, osteocalcin, renal function, lipid profile, and glycated hemoglobin.

#### 2.3.5 Nutritional assessment

Nutritional status was assessed using BMI, Mini Nutritional Assessment (MNA) total score, hemoglobin, total protein, albumin, and 25-hydroxyvitamin D. An MNA total score <24 was defined as malnutrition risk, including both risk of malnutrition and established malnutrition (7). In multivariable regression models, MNA total score was further included to evaluate whether nutritional status influenced the association between sarcopenia and fall history.

### 2.4 Statistical analysis

Continuous variables were expressed as mean ± standard deviation or median with interquartile range, depending on their distribution. Categorical variables were expressed as numbers and percentages. Between-group comparisons were performed using Student’s t-test for normally distributed continuous variables, the Mann–Whitney U test for non-normally distributed continuous variables, and the chi-square test or Fisher’s exact test for categorical variables, as appropriate. All tests were two-sided, and P values <0.05 were considered statistically significant.

First, participants were divided into sarcopenia and non-sarcopenia groups. Demographic characteristics, anthropometric indicators, sarcopenia-related measurements, fall history, nutritional status, bone metabolism, bone mineral density, and medication use were compared between the two groups. Second, participants were divided according to the presence or absence of fall history, and sarcopenia status, muscle mass, handgrip strength, gait speed, calf circumference, nutritional status, bone health, comorbidity burden, and medication burden were compared between groups.

Binary logistic regression was used to evaluate the association between sarcopenia and fall history. Fall history was used as the dependent variable, and sarcopenia status was the main independent variable, with participants without sarcopenia as the reference group. A series of progressively adjusted models was constructed as follows: the crude model included no covariates; Model 1 was adjusted for age and sex; Model 2 was further adjusted for BMI; Model 3 was further adjusted for number of comorbidities and polypharmacy; Model 4 was further adjusted for MNA total score; and Model 5 was further adjusted for osteoporosis status on the basis of Model 4. Results were presented as odds ratios (ORs) with 95% confidence intervals (CIs). Because this is a cross-sectional analysis in which sarcopenia status was assessed at a single time point after the reported fall history had already occurred, this design cannot establish the temporal or causal direction of the association: reverse causation is plausible, whereby previous falls may have led to reduced physical activity and subsequent muscle loss, rather than sarcopenia causing the falls. A complete-case (listwise deletion) approach was used for all multivariable models; no imputation was performed for missing covariate data.

To further explore the association between individual sarcopenia-related components and fall history, ASMI, low muscle mass, handgrip strength, low handgrip strength, gait speed, low gait speed, and calf circumference were separately entered into logistic regression models. Each component was analyzed in a separate model to reduce collinearity. These component models were adjusted for age, sex, BMI, number of comorbidities, and polypharmacy. For continuous variables, ORs were expressed per decrease in the specified unit when appropriate. These seven component analyses were exploratory and hypothesis-generating; results were not corrected for multiple comparisons in the primary analysis, and sensitivity analyses using Bonferroni and false discovery rate (FDR) corrections are reported in the Results.

## 3 Results

### 3.1 Study population

**Figure 1:**
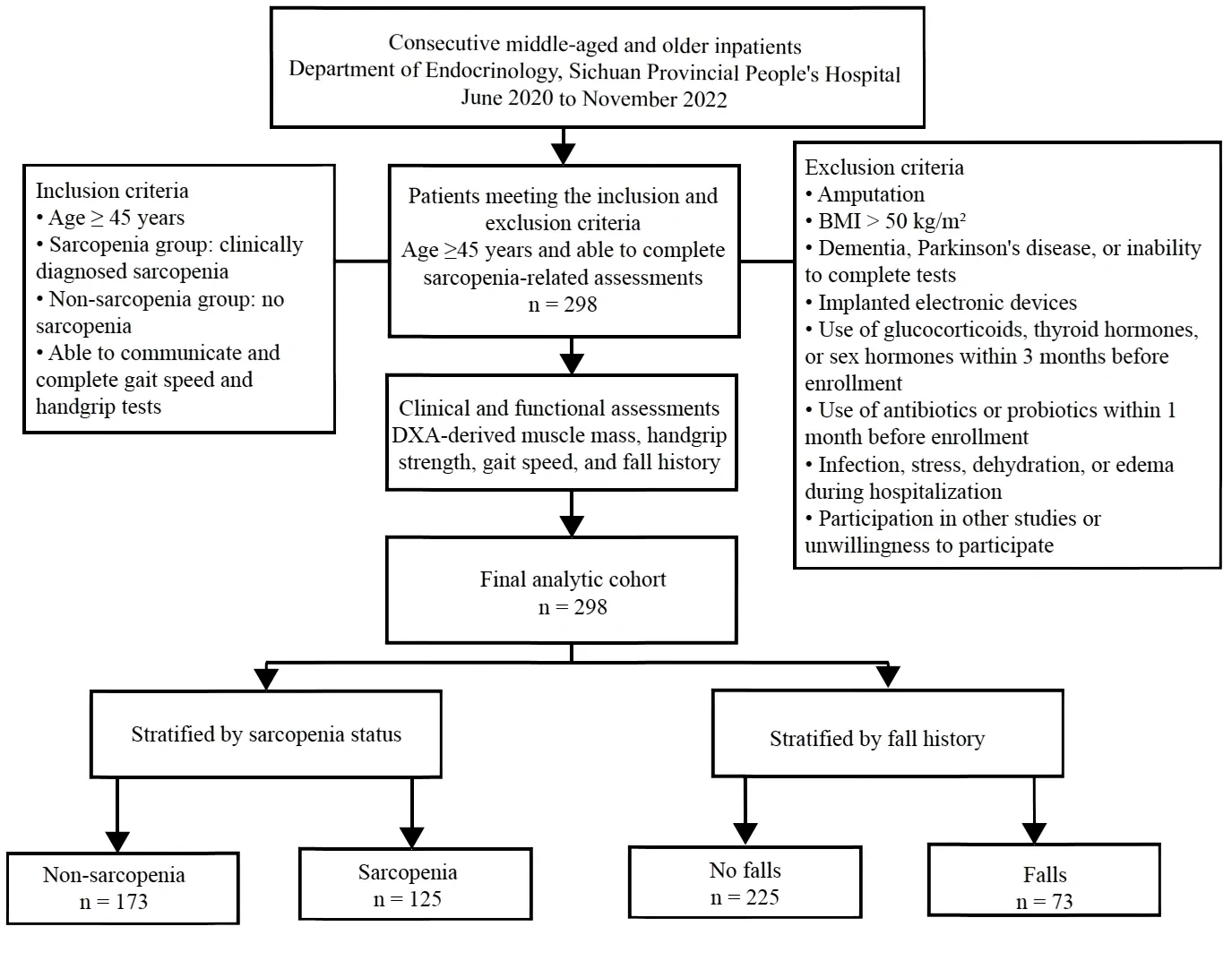
Flowchart of participant selection and study grouping.

**Figure 2:**
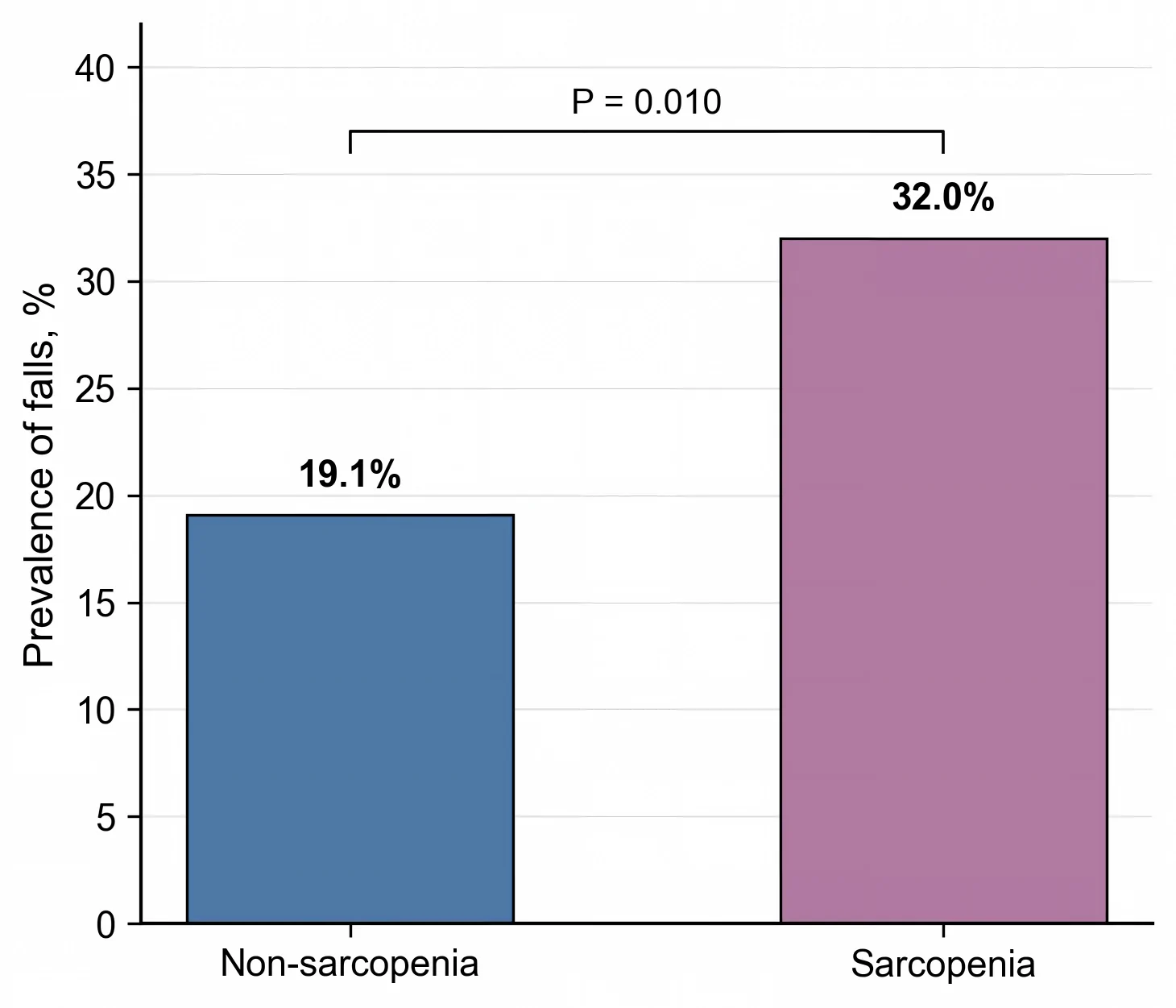
Prevalence of fall history by sarcopenia status.

A total of 298 participants were included in the final analysis. Among them, 173 participants were classified as non-sarcopenic and 125 as sarcopenic. Overall, 73 participants had a history of falls, corresponding to a fall history prevalence of 24.5%. The participant selection process and study grouping are shown in **Figure 1**. The prevalence of fall history according to sarcopenia status is shown in **Figure 2**.

### 3.2 Baseline characteristics according to sarcopenia status

The baseline clinical characteristics of participants stratified by sarcopenia status are presented in **Table 1**. Compared with participants without sarcopenia, those with sarcopenia had a significantly higher proportion of men (40.0% vs. 13.9%, P<0.001) and a lower BMI (21.59 ± 3.05 vs. 24.39 ± 3.06 kg/m², P<0.001). Age and education level did not differ significantly between the two groups.

**Table 1:** Baseline clinical characteristics of participants stratified by sarcopenia status.

Variables	Non-sarcopenia (n=173)	Sarcopenia (n=125)	P-value
Demographic and anthropometric characteristics			
Age, years	68.76 ± 8.35	70.00 ± 7.81	0.191
Sex, Male, n (%)	24 (13.9%)	50 (40.0%)	<0.001
Education ≤ 9 years, n (%)	88 (51.2%)	66 (53.2%)	0.726
BMI, kg/m²	24.39 ± 3.06	21.59 ± 3.05	<0.001
Fall- and fracture-related characteristics			
History of falls, n (%)	33 (19.1%)	40 (32.0%)	0.010
Fragility fracture after age 45, n (%)	60 (34.7%)	37 (29.8%)	0.380
Height loss ≥3 cm, n (%)	86 (50.0%)	65 (52.4%)	0.681
Pain score	2.00 (1.00, 4.00)	2.00 (0.00, 4.00)	0.319
Sarcopenia-related measurements			
ASMI, kg/m²	6.25 ± 0.67	5.50 ± 0.81	<0.001
Handgrip strength, kg	21.20 (17.70, 25.58)	20.30 (16.55, 25.60)	0.457
Low handgrip strength, n (%)	46 (27.1%)	57 (47.9%)	<0.001
Gait speed, m/s	0.95 (0.78, 1.09)	0.85 (0.66, 1.01)	0.002
Low gait speed <1.0 m/s, n (%)	95 (55.9%)	81 (69.2%)	0.023
Mid-upper arm circumference, cm	27.70 (25.50, 30.00)	25.00 (23.00, 28.00)	<0.001
Calf circumference, cm	33.00 (31.00, 35.00)	31.00 (29.25, 32.75)	<0.001
Comprehensive geriatric assessment and medication burden			
Number of comorbidities	7.00 (5.00, 9.00)	7.00 (5.00, 10.00)	0.574
Polypharmacy ≥6 medications, n (%)	99 (57.2%)	82 (65.6%)	0.144
MNA total score	23.00 (20.50, 24.50)	20.25 (16.50, 22.50)	<0.001
Malnutrition risk (MNA <24), n (%)	116 (67.4%)	106 (86.9%)	<0.001
Nutritional and inflammatory markers			
Hemoglobin, g/L	127.00 (119.00, 135.50)	128.00 (115.00, 141.00)	0.557
Albumin, g/L	41.80 (39.25, 43.80)	39.85 (37.10, 42.42)	<0.001
25-hydroxyvitamin D, ng/mL	20.50 (15.62, 27.68)	20.87 (15.74, 27.21)	0.792
C-reactive protein, mg/L	0.96 (0.50, 2.01)	1.46 (0.61, 6.85)	0.002
Bone metabolism and bone health			
Serum calcium, mmol/L	2.33 (2.24, 2.43)	2.30 (2.18, 2.38)	0.014
Serum phosphate, mmol/L	1.16 (1.02, 1.28)	1.12 (1.00, 1.29)	0.321
Alkaline phosphatase, U/L	78.00 (64.00, 103.00)	82.00 (67.50, 103.00)	0.330
Osteoporosis, n (%)	72 (47.7%)	62 (57.4%)	0.123
Parathyroid hormone, pg/mL	40.40 (27.73, 61.27)	40.70 (27.00, 58.80)	0.799
Femoral neck T-score	-1.80 (-2.40, -1.10)	-2.10 (-2.80, -1.30)	0.026
Total hip T-score	-1.55 (-2.23, -0.60)	-1.90 (-2.60, -0.93)	0.033
L1–L4 T-score	-2.10 (-2.98, -0.93)	-2.25 (-3.10, -0.10)	0.845
Treatment-related factors			
Calcium supplementation, n (%)	119 (68.8%)	75 (60.5%)	0.138
Vitamin D supplementation, n (%)	68 (39.3%)	43 (34.4%)	0.387
Anti-osteoporosis medication, n (%)	47 (27.2%)	32 (25.6%)	0.762

Data are expressed as mean ± SD, median (IQR), or n (%), as appropriate. Percentages were calculated using the number of participants with available data for each variable as the denominator. P-values were derived from Student’s t-test, Mann–Whitney U test, χ² test, or Fisher’s exact test, as appropriate. ASMI, appendicular skeletal muscle mass index; BMI, body mass index; MNA, Mini Nutritional Assessment.

Participants with sarcopenia had a higher prevalence of fall history than those without sarcopenia (32.0% vs. 19.1%, P = 0.010). However, there were no significant between-group differences in fragility fracture after age 45, height loss ≥3 cm, or pain score.

As expected, sarcopenia-related measurements differed substantially between groups. Participants with sarcopenia had significantly lower ASMI, slower gait speed, smaller mid-upper arm circumference, and smaller calf circumference. The prevalence of low handgrip strength and low gait speed was also significantly higher in the sarcopenia group. However, handgrip strength as a continuous variable did not differ significantly between groups.

Regarding comprehensive geriatric assessment and medication burden, the number of comorbidities and the prevalence of polypharmacy did not differ significantly between groups. In contrast, participants with sarcopenia had a significantly lower MNA total score and a higher prevalence of malnutrition risk than those without sarcopenia.

For nutritional and inflammatory markers, the sarcopenia group had lower albumin levels and higher C-reactive protein levels. Hemoglobin and 25-hydroxyvitamin D levels did not differ significantly between groups. In terms of bone health, femoral neck and total hip T-scores were significantly lower in participants with sarcopenia, whereas the prevalence of osteoporosis and L1–L4 T-score did not differ significantly between groups. Calcium supplementation, vitamin D supplementation, and anti-osteoporosis medication use were also comparable between groups.

### 3.3 Clinical characteristics according to fall history

The clinical characteristics of participants according to fall history are shown in **Table 2**. Participants with fall history had a significantly higher prevalence of sarcopenia than those without fall history (54.8% vs. 37.8%, P = 0.010). Age, education level, and BMI were not significantly different between the two groups. The proportion of men was lower among participants with fall history, although the difference did not reach statistical significance (16.4% vs. 27.6%, P = 0.056).

**Table 2:** Clinical characteristics according to fall history.

Variable	No falls (n=225)	Falls (n=73)	P-value
Demographic and anthropometric characteristics			
Age, years	69.00 (64.00, 75.00)	69.00 (64.00, 77.00)	0.804
Sex, Male, n (%)	62/225 (27.6%)	12/73 (16.4%)	0.056
Education ≤9 years, n (%)	117/224 (52.2%)	37/72 (51.4%)	0.901
BMI, kg/m²	23.11 (20.92, 25.30)	22.28 (20.58, 24.45)	0.133
Fall- and fracture-related characteristics			
Fragility fracture after age 45, n (%)	68/225 (30.2%)	29/72 (40.3%)	0.113
Height loss ≥3 cm, n (%)	115/224 (51.3%)	36/72 (50.0%)	0.843
Pain score	2.00 (0.00, 4.00)	2.00 (1.00, 4.50)	0.180
Sarcopenia-related measurements			
Sarcopenia, n (%)	85/225 (37.8%)	40/73 (54.8%)	0.010
ASMI, kg/m²	6.03 ± 0.83	5.63 ± 0.69	<0.001
Handgrip strength, kg	21.15 (17.62, 26.05)	19.90 (16.10, 23.70)	0.072
Gait speed, m/s	0.92 ± 0.27	0.87 ± 0.27	0.156
Mid-upper arm circumference, cm	27.00 (25.00, 29.00)	26.00 (23.00, 29.00)	0.021
Calf circumference, cm	33.00 (30.50, 35.00)	31.00 (30.00, 33.00)	0.002
Comprehensive geriatric assessment and medication burden			
Number of comorbidities	6.00 (4.00, 9.00)	7.00 (5.00, 10.00)	0.066
Polypharmacy ≥6 medications, n (%)	136/225 (60.4%)	45/73 (61.6%)	0.855
MNA total score	22.00 (19.00, 24.00)	21.00 (19.00, 23.25)	0.276
Malnutrition risk (MNA <24), n (%)	164/223 (73.5%)	58/71 (81.7%)	0.164
Nutritional and inflammatory markers			
Hemoglobin, g/L	128.00 (118.50, 139.00)	126.00 (113.00, 133.00)	0.034
Albumin, g/L	41.10 (38.70, 43.40)	40.00 (37.60, 43.20)	0.163
25-hydroxyvitamin D, ng/mL	20.66 (15.70, 26.97)	20.05 (15.18, 29.45)	0.669
C-reactive protein, mg/L	1.06 (0.50, 2.51)	1.26 (0.50, 2.76)	0.669
Bone metabolism and bone health			
Serum calcium, mmol/L	2.31 (2.22, 2.42)	2.31 (2.20, 2.42)	0.693
Serum phosphate, mmol/L	1.14 (1.00, 1.29)	1.16 (1.03, 1.26)	0.870
Alkaline phosphatase, U/L	80.00 (64.25, 102.75)	83.00 (70.00, 103.00)	0.626
Osteoporosis, n (%)	106/198 (53.5%)	28/61 (45.9%)	0.297
Parathyroid hormone, pg/mL	40.60 (28.30, 60.85)	40.40 (24.23, 60.17)	0.596
Femoral neck T-score	-1.90 (-2.68, -1.12)	-1.90 (-2.50, -1.30)	0.870
Total hip T-score	-1.60 (-2.40, -0.60)	-1.65 (-2.42, -1.00)	0.303
L1–L4 T-score	-2.10 (-3.10, -0.60)	-2.20 (-2.90, -1.00)	0.800
Treatment-related factors.			
Calcium supplementation, n (%)	141/224 (62.9%)	53/73 (72.6%)	0.132
Vitamin D supplementation, n (%)	75/225 (33.3%)	36/73 (49.3%)	0.014
Anti-osteoporosis medication, n (%)	56/225 (24.9%)	23/73 (31.5%)	0.266

Data are expressed as mean ± SD, median (IQR), or n/N (%), as appropriate. Percentages were calculated using the number of participants with available data for each variable as the denominator. For osteoporosis and site-specific T-scores, this denominator reflects only the subset of participants with available bone mineral density data (n=198 for the no-falls group and n=61 for the falls group; total n=259/298), which differs from the full-cohort denominators used for the sarcopenia-status comparisons in **Table 1**. P-values were derived from Student’s t-test, Mann–Whitney U test, χ² test, or Fisher’s exact test, as appropriate. Malnutrition risk was defined as MNA <24. Osteoporosis was defined as a T-score ≤−2.5 at the femoral neck, total hip, or lumbar spine. ASMI, appendicular skeletal muscle mass index; BMI, body mass index; MNA, Mini Nutritional Assessment.

With respect to sarcopenia-related measurements, participants with fall history had significantly lower ASMI than those without fall history (5.63 ± 0.69 vs. 6.03 ± 0.83 kg/m², P<0.001). Mid-upper arm circumference and calf circumference were also significantly lower in the fall history group. Handgrip strength and gait speed tended to be lower among participants with fall history, but these differences were not statistically significant.

The number of comorbidities was slightly higher in participants with fall history, but the difference did not reach statistical significance. The prevalence of polypharmacy was similar between groups. MNA total score and malnutrition risk were not significantly different according to fall history. Among laboratory markers, hemoglobin was significantly lower in participants with fall history, whereas albumin, 25-hydroxyvitamin D, and C-reactive protein did not differ significantly between groups.

Bone metabolism markers, osteoporosis prevalence, and site-specific T-scores were not significantly different between participants with and without fall history. Regarding treatment-related factors, vitamin D supplementation was more common in participants with fall history, whereas calcium supplementation and anti-osteoporosis medication use did not differ significantly between groups.

### 3.4 Association between sarcopenia and fall history

**Figure 3:**
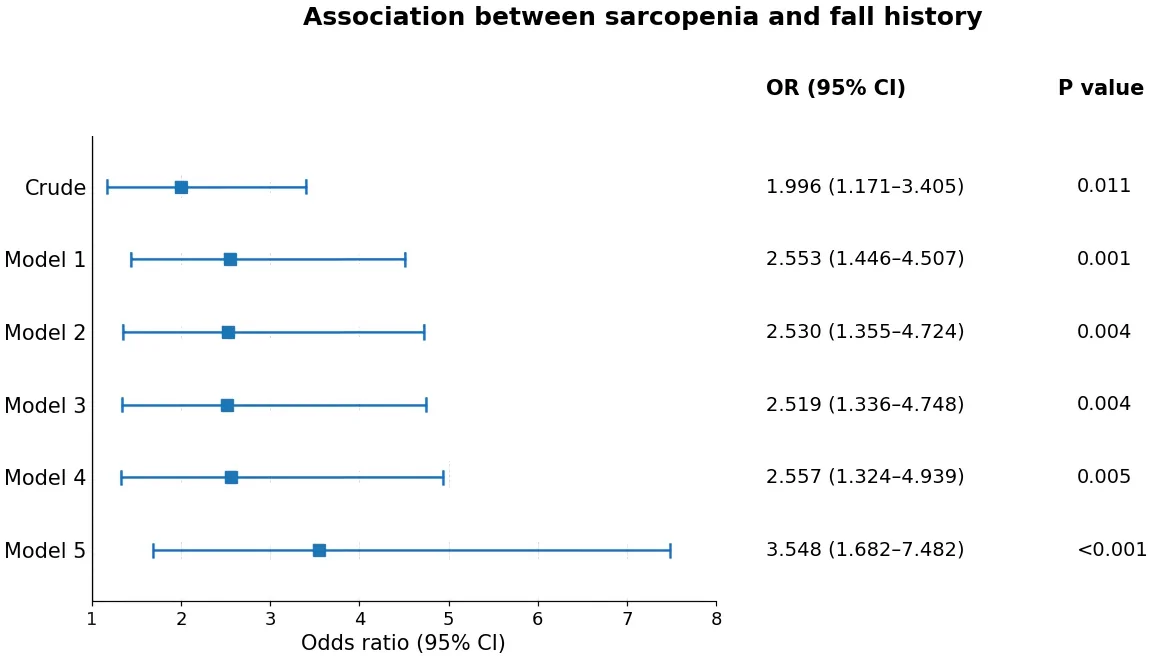
Association between sarcopenia and fall history across progressively odels.

The association between sarcopenia and fall history was evaluated using binary logistic regression models (**Table 3**; **Figure 3**). In the crude model, sarcopenia was significantly associated with fall history, with an OR of 1.996 (95% CI 1.171–3.405, P = 0.011).

**Table 3:** Association between sarcopenia and fall history.

Model	Adjusted covariates	OR (95% CI)	P value
Crude model	None	1.996 (1.171–3.405)	0.011
Model 1	Age and sex	2.553 (1.446–4.507)	0.001
Model 2	Model 1 + BMI	2.530 (1.355–4.724)	0.004
Model 3	Model 2 + comorbidities and polypharmacy	2.519 (1.336–4.748)	0.004
Model 4	Model 3 + MNA total score	2.557 (1.324–4.939)	0.005
Model 5	Model 4 + osteoporosis	3.548 (1.682–7.482)	<0.001

Data are presented as odds ratios (ORs) with 95% confidence intervals (CIs) derived from binary logistic regression models. Fall history was used as the dependent variable, and sarcopenia status was the main independent variable. The reference group was participants without sarcopenia. Events indicate participants with a history of falls. Polypharmacy was defined as the use of ≥6 medications. Osteoporosis was defined as a T-score ≤−2.5 at the femoral neck, total hip, or lumbar spine. BMI, body mass index; MNA, Mini Nutritional Assessment; OR, odds ratio; CI, confidence interval.

After adjustment for age and sex in Model 1, the association remained significant and became stronger (OR = 2.553, 95% CI 1.446–4.507, P = 0.001). Further adjustment for BMI in Model 2 did not materially change the result (OR = 2.530, 95% CI 1.355–4.724, P = 0.004). In Model 3, after additional adjustment for number of comorbidities and polypharmacy, sarcopenia remained significantly associated with fall history (OR = 2.519, 95% CI 1.336–4.748, P = 0.004).

The association also remained stable after further adjustment for MNA total score in Model 4 (OR = 2.557, 95% CI 1.324–4.939, P = 0.005). In the fully adjusted Model 5, which additionally included osteoporosis status on the basis of Model 4, sarcopenia remained significantly associated with fall history (OR = 3.548, 95% CI 1.682–7.482, P<0.001). This fully adjusted model included 257 participants with available data, among whom 61 had fall history.

### 3.5 Association of sarcopenia-related components with fall history

The associations between individual sarcopenia-related components and fall history are presented in **Table 4**. After adjustment for age, sex, BMI, number of comorbidities, and polypharmacy, lower ASMI was significantly associated with fall history. For each 1 kg/m² decrease in ASMI, the odds of fall history increased by approximately 95% (OR = 1.948, 95% CI 1.190–3.191, P = 0.008).

**Table 4:** Association of sarcopenia-related components with fall history.

Variable	OR	95% CI	P value
ASMI, per 1 kg/m² decrease	1.948	1.190–3.191	0.008
Low muscle mass	2.799	1.467–5.340	0.002
Handgrip strength, per 1 kg increase	1.004	0.962–1.048	0.848
Low handgrip strength	1.172	0.637–2.155	0.610
Gait speed, per 0.1 m/s decrease	1.066	0.953–1.193	0.261
Low gait speed	1.308	0.713–2.400	0.385
Calf circumference, per 1 cm decrease	1.180	1.049–1.326	0.006

ORs and 95% CIs were derived from binary logistic regression models adjusted for age, sex, BMI, number of comorbidities, and polypharmacy. Each sarcopenia-related component was analyzed in a separate model. For continuous variables, ORs were expressed per decrease in the specified unit, except for handgrip strength, which was expressed per 1 kg increase. ASMI, appendicular skeletal muscle mass index; BMI, body mass index; CI, confidence interval; OR, odds ratio.

Low muscle mass was also significantly associated with fall history (OR = 2.799, 95% CI 1.467–5.340, P = 0.002). Calf circumference showed a significant inverse association with fall history; each 1 cm decrease in calf circumference was associated with higher odds of fall history (OR = 1.180, 95% CI 1.049–1.326, P = 0.006).

In contrast, handgrip strength, low handgrip strength, gait speed, and low gait speed were not significantly associated with fall history in the adjusted models. Because these component analyses were exploratory and uncorrected for multiple comparisons, we additionally applied Bonferroni correction (adjusted α=0.05/7 = 0.0071) and the Benjamini–Hochberg false discovery rate (FDR) procedure. Under FDR correction, all three components originally reported as significant remained significant (low muscle mass, P = 0.002; calf circumference, P = 0.006; ASMI, P = 0.008); under the more conservative Bonferroni correction, low muscle mass and calf circumference remained significant, whereas the association for ASMI narrowly did not survive the corrected threshold, and should therefore be interpreted with corresponding caution.

### 3.6 Exploratory bone–muscle phenotype analysis

An exploratory analysis was conducted among participants with available bone mineral density data to examine fall history across bone–muscle phenotypes. The prevalence of fall history was 21.5% in the normal group, 13.9% in the osteoporosis-only group, 34.8% in the sarcopenia-only group, and 29.0% in the sarcopenia plus osteoporosis group.

Participants with both sarcopenia and osteoporosis had the lowest MNA total score among the four groups, suggesting a greater nutritional vulnerability in this combined phenotype. However, fall history was more frequent in the sarcopenia-related groups than in the osteoporosis-only group, supporting the observation that fall history in this cohort was more closely related to sarcopenia and reduced muscle reserve than to osteoporosis alone. This analysis was exploratory and is presented in **Supplementary Table 1**. This analysis was exploratory and intended to characterize phenotype-level patterns descriptively; formal between-group significance testing was not performed for these subgroup comparisons.

## 4 Discussion

In this cross-sectional clinical study of middle-aged and older adults, we found that sarcopenia was significantly associated with fall history. Participants with sarcopenia had a higher prevalence of fall history than those without sarcopenia, and participants with fall history showed a higher prevalence of sarcopenia than those without fall history. This association remained significant after progressive adjustment for age, sex, BMI, comorbidity burden, polypharmacy, nutritional status, and osteoporosis, suggesting that the relationship between sarcopenia and fall history was not fully explained by these clinical factors. In further component analyses, lower ASMI, low muscle mass, and smaller calf circumference were significantly associated with fall history, whereas handgrip strength and gait speed were not. These findings indicate that reduced muscle reserve, particularly low muscle mass and decreased calf circumference, may be especially relevant to fall history in this clinical population.

Our findings are consistent with previous evidence showing that sarcopenia is associated with an increased risk of falls and fractures in older adults. Meta-analyses have reported that older adults with sarcopenia have a higher risk of falls than those without sarcopenia (3, 4), and recent evidence from Chinese older adults has further supported the relevance of sarcopenia to fall-risk assessment in Asian populations (9). Compared with many population-based studies, the present study included detailed clinical measurements, including DXA-derived muscle mass, handgrip strength, gait speed, calf circumference, nutritional assessment, bone mineral density, comorbidity burden, and medication burden. Therefore, our findings extend previous observations by showing that the association between sarcopenia and fall history persists after accounting for these additional clinical factors.

A notable finding of this study was that muscle reserve-related indicators were more clearly associated with fall history than single functional measurements. In the component analysis, lower ASMI, low muscle mass, and smaller calf circumference were significantly associated with fall history, whereas handgrip strength, low handgrip strength, gait speed, and low gait speed were not. This pattern may partly reflect the nature of fall history as a previous event, whereas handgrip strength and gait speed were measured only at the time of assessment. In contrast, ASMI and calf circumference may better reflect relatively stable muscle reserve, nutritional reserve, and long-term physical status. Although some previous studies have reported stronger associations between gait speed or muscle strength and falls (10), differences in study design, population characteristics, outcome definition, and timing of assessment may partly explain these discrepancies. In addition, because all participants were hospitalized inpatients, acute illness, bed rest, and the physiological stress of hospitalization may have transiently impaired gait speed and handgrip strength at the time of assessment, independent of each participant’s usual functional status. This inpatient-setting effect could partly explain why these performance-based measures, which reflect status at a single time point, showed weaker associations with fall history over the preceding 12 months than more stable indicators of muscle reserve such as ASMI and calf circumference, which are less susceptible to short-term perturbation by acute hospitalization.

Calf circumference may be particularly useful in routine clinical practice. The AWGS 2019 consensus recommends calf circumference as a practical case-finding tool for sarcopenia in Asian populations (2). In the present study, smaller calf circumference was significantly associated with fall history after adjustment for major confounders. Because calf circumference is inexpensive, noninvasive, and easy to measure, it may help identify individuals who require further assessment for sarcopenia and fall risk, especially in settings where DXA or bioelectrical impedance analysis is not readily available. Studies in Chinese older adults have also supported calf circumference as a useful screening tool for sarcopenia (11, 12).

Nutritional impairment is closely related to sarcopenia and may contribute to reduced muscle reserve and overall vulnerability. In this study, participants with sarcopenia had lower MNA total scores, a higher prevalence of malnutrition risk, lower albumin levels, and higher C-reactive protein levels than those without sarcopenia. These findings suggest that sarcopenia was accompanied by poorer nutritional status and a greater inflammatory burden. Previous studies have also reported associations between poor nutritional status and sarcopenia in older adults (5), and chronic low-grade inflammation has been linked to muscle wasting and sarcopenia (13). However, nutritional and inflammatory markers did not differ significantly between participants with and without fall history, and the association between sarcopenia and fall history remained significant after adjustment for MNA total score. Therefore, nutritional impairment should be interpreted as an important clinical background of sarcopenia-related vulnerability rather than as a confirmed mediator between sarcopenia and falls.

Bone health is another important dimension in the assessment of older adults with sarcopenia. In the present study, participants with sarcopenia had lower femoral neck and total hip T-scores, suggesting poorer hip bone health. However, osteoporosis prevalence and site-specific T-scores did not differ significantly between participants with and without fall history. This suggests that osteoporosis alone did not explain fall history in this cohort. Clinically, bone mineral density may be more directly related to fracture risk after a fall, whereas fall occurrence itself is influenced by muscle reserve, balance, gait, cognition, medications, comorbidities, and environmental factors. The exploratory bone–muscle phenotype analysis also showed that fall history was more frequent in the sarcopenia-related groups than in the osteoporosis-only group, supporting the interpretation that fall history was more closely linked to sarcopenia and reduced muscle reserve than to osteoporosis alone. Nevertheless, this exploratory analysis was limited to participants with available bone mineral density data and should be interpreted cautiously. Recent evidence also suggests that osteosarcopenia may not always confer a clearly higher fall risk than sarcopenia alone (14).

Comorbidity burden and polypharmacy are established components of fall-risk assessment in older adults. Current fall prevention guidelines emphasize that fall risk should be evaluated using a multidomain approach, including medications, comorbidities, gait and balance, cognition, nutrition, and fracture risk (1). Polypharmacy may increase fall risk through adverse effects such as dizziness, orthostatic hypotension, sedation, hypoglycemia, or electrolyte disturbance. At the same time, medication burden may also reflect multimorbidity and overall frailty. Morin et al. reported that the association between polypharmacy and injurious falls was attenuated after adjustment for chronic disease burden and fall-risk-increasing medications, highlighting the complexity of this relationship (6). In the present study, sarcopenia remained significantly associated with fall history after adjustment for comorbidity burden and polypharmacy, suggesting that sarcopenia may provide fall-risk information beyond comorbidity and medication burden alone.

**Figure 4:**
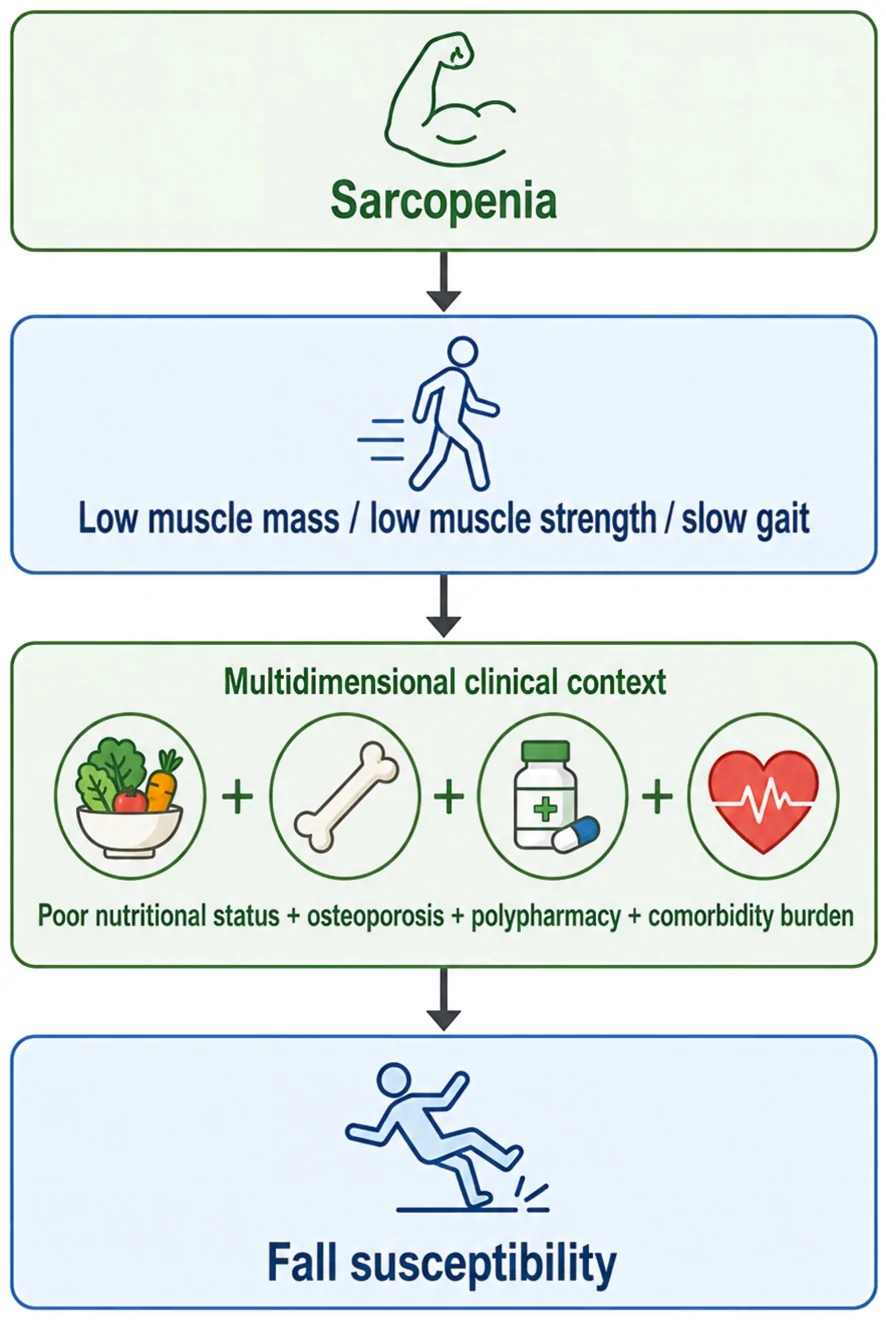
Conceptual framework of sarcopenia-related fall susceptibility. This image illustrates the multidimensional clinical context of sarcopenia-related fall susceptibility and should not be interpreted as a causal mediation pathway.

The findings of this study have practical clinical implications. Patients with sarcopenia should be considered a priority population for fall-risk screening. In routine practice, assessment should not be limited to age, osteoporosis, or previous fall history, but should also include muscle reserve, calf circumference, nutritional status, comorbidity burden, and medication review. For patients with sarcopenia, especially those with low ASMI or reduced calf circumference, comprehensive interventions including nutritional support, resistance exercise, balance training, medication review, comorbidity management, and bone health optimization may be considered. The conceptual framework shown in **Figure 4** summarizes these interrelated clinical domains; however, it should be interpreted as an illustrative model rather than as a causal mediation pathway.

The major strength of this study is the availability of comprehensive clinical assessment data, including DXA-derived muscle mass and bone mineral density, handgrip strength, gait speed, calf circumference, nutritional assessment, laboratory markers, comorbidity burden, medication burden, and bone health indicators. This allowed us to evaluate the association between sarcopenia and fall history while accounting for these additional clinical dimensions. Nevertheless, several limitations should be acknowledged. First, the cross-sectional design precludes causal inference. Although sarcopenia was associated with fall history, we cannot determine whether sarcopenia preceded falls or whether previous falls contributed to reduced activity and subsequent muscle loss. Second, fall history was based on previous records or self-report and may be subject to recall bias. Third, participants were recruited exclusively from hospitalized inpatients at a single endocrinology department, and individuals with dementia, Parkinson’s disease, and other conditions strongly associated with elevated fall risk were excluded; our findings therefore reflect a hospitalized, multimorbid clinical population rather than a general community-dwelling older adult population, and the generalizability of our findings should be considered limited until confirmed in community-based cohorts. Fourth, some variables, including bone mineral density, were missing in a subset of participants. We used a complete-case (listwise deletion) approach for all multivariable models. A sensitivity comparison between participants included in and excluded from the fully adjusted Model 5 (n=257 included vs. n=41 excluded) showed no significant differences in age, sex, BMI, sarcopenia status, fall history, polypharmacy, or MNA total score (all P>0.05), although excluded participants had a significantly higher number of comorbidities (9.37 vs. 6.76, P = 0.001), suggesting that missingness, largely reflecting bone mineral density data availability, was not strongly related to the primary exposure or outcome. Fifth, detailed information on fall timing, frequency, location, severity, and fall-related injuries was unavailable. Sixth, the fully adjusted Model 5 included 61 fall events across 8 covariates (events-per-variable ratio ≈7.6), below the conventional threshold of 10; although simulation studies suggest EPV values of 5–9 yield acceptable bias in logistic regression (15), this reduced ratio may widen confidence intervals and increase the risk of overfitting, and the fully adjusted estimates should be interpreted with corresponding caution. Seventh, calf circumference was measured only on the right leg, and potential left-right asymmetry (e.g., due to unilateral joint disease, prior lower-limb fracture, or hemiparesis) was not assessed. Eighth, handgrip strength was measured in the standing position rather than the seated, elbow-at-90° position recommended by AWGS 2019, which may have introduced postural-stability-related confounding into this measurement. Finally, medication burden was assessed using polypharmacy rather than specific fall-risk-increasing drug classes, and important fall-related factors such as balance function, cognition, vision, environmental hazards, and fear of falling were not fully assessed.

## 5 Conclusion

In conclusion, sarcopenia was significantly associated with fall history in middle-aged and older adults, and this association persisted after adjustment for age, sex, BMI, comorbidity burden, polypharmacy, nutritional status, and osteoporosis. Among sarcopenia-related components, low muscle mass and smaller calf circumference showed the most evident associations with fall history, whereas handgrip strength and gait speed were not significantly associated in this cohort. These findings suggest that reduced muscle reserve may be an important clinical marker of fall susceptibility. Fall-risk assessment in individuals with sarcopenia should therefore be conducted within a multidimensional clinical framework that incorporates muscle reserve, nutritional status, bone health, comorbidity burden, and medication burden.

## Data Availability

The raw data supporting the conclusions of this article will be made available by the authors, without undue reservation.
