# Supplementary material for: Association between sarcopenia and fall history in middle-aged and older adults: a multidimensional clinical analysis of nutritional status, bone health, and medication burden
Source: Front Endocrinol (Lausanne). 2026 Aug 12;17:1906385. doi: 10.3389/fendo.2026.1906385 (PMC13506441; doi:10.3389/fendo.2026.1906385)
Supplement: Supplementary file 1 [file Table1.docx]

Supplementary Table 1. Exploratory bone-muscle phenotypes according to sarcopenia and osteoporosis status

| **Group** | **n** | **Falls, n (%)** | **MNA** | **Polypharmacy, n (%)** | **Lowest T-score** |
| --- | --- | --- | --- | --- | --- |
| Normal | 79 | 17 (21.5%) | 23.00 (20.50, 24.75) | 47 (59.5%) | -1.60 (-2.00, -0.90) |
| Osteoporosis only | 72 | 10 (13.9%) | 22.00 (20.75, 23.75) | 39 (54.2%) | -3.10 (-3.50, -2.80) |
| Sarcopenia only | 46 | 16 (34.8%) | 21.50 (20.00, 23.00) | 29 (63.0%) | -1.25 (-1.98, -0.60) |
| Sarcopenia + osteoporosis | 62 | 18 (29.0%) | 19.25 (15.12, 22.38) | 37 (59.7%) | -3.10 (-3.68, -2.80) |

Data are expressed as n, n (%), or median (IQR), as appropriate. Osteoporosis was defined as a T-score ≤ -2.5 at the femoral neck, total hip, or lumbar spine. The lowest T-score was calculated as the lowest available T-score among the femoral neck, total hip, and L1-L4. This exploratory analysis included participants with available bone mineral density data. MNA, Mini Nutritional Assessment.
